# Ophthalmic artery changes in type 2 diabetes with and without acute coronary syndrome

**DOI:** 10.1186/s12967-022-03712-0

**Published:** 2022-11-05

**Authors:** Lan-ting Wu, Jia-lin Wang, Yan-ling Wang

**Affiliations:** Department of Ophthalmology, Beijing Friendship Hospital, Capital Medical University, Beijing, 100050 China

**Keywords:** Acute coronary syndrome, Computational fluid dynamics, Hemodynamic numerical simulation, Ophthalmic artery, Type 2 diabetes

## Abstract

**Background:**

Ocular blood flow provides a new perspective for studying the effects of diabetes and ischemic heart disease on systemic blood flow, pathological mechanisms, and prognosis. Previous studies have analyzed the hemodynamic changes of the ophthalmic artery (OA) in patients with diabetes and ischemic heart disease, but the results remain controversial due to limited observation methods. We aimed to explore the morphological and hemodynamic features in the OA in patients with type 2 diabetes (T2D) with and without acute coronary syndrome (ACS).

**Methods:**

In total, 134 participants, including 30 control participants, 34 with ACS only, 34 with T2D only, and 36 with both ACS and T2D, undergoing computed tomography angiography were enrolled. Three-dimensional OA models were reconstructed, and morphological parameters of the OA were measured. In addition, numerical simulations using computational fluid dynamics were used to acquire hemodynamic parameters of the OA.

**Results:**

In this study, 134 OA models were reconstructed. Morphological measurements revealed a smaller initial OA diameter in the T2D group than in the other two ACS groups. A hemodynamic simulation showed a significantly lower OA blood velocity in patients with ACS and T2D than that in controls (*P* < 0.001). The mass flow ratios in all disease groups were lower than those in the control group (*P* < 0.001, *P* = 0.020, and *P* < 0.001, respectively). The ACS and T2D groups had higher OA pressure levels than those of the control group (*P* = 0.013). The OA blood velocity and mass flow ratio were correlated with several clinical parameters.

**Conclusions:**

This study revealed morphological and hemodynamic differences in the OA between patients with T2D with and without ACS. Furthermore, the hemodynamic characteristics of the OA correlated with clinical prognostic biomarkers, suggesting the potential predictive ability of the OA.

## Background

Cardiovascular disease is a common comorbidity of type 2 diabetes (T2D), affecting approximately one-third of patients with T2D worldwide [1]. In addition to being a risk factor for the development of coronary heart disease, diabetes also affects the prognosis of acute coronary syndrome (ACS) [2]. Zhou et al. showed that patients with ACS and diabetes had a two-fold increased risk of all-cause death and a 1.5-fold increased risk of major adverse cardiovascular and cerebrovascular events, compared to patients without diabetes [3]. Macrovascular and microvascular complications are common in patients with diabetes. Studies have analyzed the effects of diabetes and ischemic heart disease on systemic blood flow, pathological mechanisms, and prognosis from the perspective of ocular blood flow [4–6]. Most of these studies are based on the retinal vascular system, because of its clinical observability. In vitro studies have shown that diabetes reduces retinal Connexin 43 (Cx43) expression, and the development of pericyte loss and acellular capillaries is associated with decreased Cx43 expression in diabetic retinopathy [7]. However, retinal vessels are affected by many confounding factors, such as age, sex, blood pressure, blood lipids, smoking, drinking, and drugs, which are difficult to completely exclude during research [8].

The ophthalmic artery (OA), as the origin of the blood supply to the eye, can directly and accurately reflect relatively early changes in blood supply. At present, most studies have used color Doppler imaging (CDI) to observe hemodynamic changes in the OA. Most of these studies concluded that the resistive index was significantly higher in patients with T2D, while other hemodynamic changes were inconclusive [9–11].This may be related to the fact that the results measured by CDI are significantly affected by human factors [12]. Changes in patient position during the CDI examination, examiners’ operating pressure, and the probe position may influence hemodynamic results. It is difficult for CDI to locate the origin of the OA or display the full length due to the course variability and small diameter of the OA. In recent years, blood simulations based on computational fluid dynamics (CFD) have been widely used in the hemodynamic analysis of cardiovascular diseases [13–15]. CFD numerical simulations based on computed tomography angiography (CTA), which can accurately obtain hemodynamic information on diseased vessels and reflect the real blood flow status in the body.

We previously reconstructed three-dimensional (3D) OA models and adopted CFD numerical simulations to examine the morphological and hemodynamic features of OA in patients with ACS and found a slower blood velocity [16]. Based on the previous study, the current study aimed to explore the morphological and hemodynamic characteristics of the OA in patients with T2D, with or without ACS, and to assess the correlation between OA characteristics and clinical indicators.

## Methods

### Study population

This was a cross-sectional study of OA characteristics in patients with T2D, with and without ACS. This study (ChiCTR2100050428) adhered to the tenets of the Declaration of Helsinki and was approved by the local ethics committee of the Beijing Friendship Hospital (2020-P2-008-01). All the participants provided written informed consent.

Four groups were defined, prior to recruitment: control participants, patients with ACS without T2D, patients with T2D without ACS, and patients with both ACS and T2D. We reviewed the electronic medical records of patients with ACS and T2D who underwent head and neck CTA in Beijing Friendship Hospital from September 2021 to February 2022 and of healthy individuals who underwent CTA for other reasons. The CTA acquisition method was the same as that in our previous study [16]. All patients had a clear diagnosis. We reviewed the hospital electronic medical records and collected and recorded the relevant clinical parameters. All participants underwent detailed ophthalmic examination, including Snellen best-corrected visual acuity, intraocular pressure (by noncontact tonometry), and slit-lamp examinations. The exclusion criteria were: (1) ocular refractive medium opacity, orbital space-occupying disease, glaucoma, optic neuritis, or other evident ocular lesions; (2) intracranial space-occupying lesions or intracranial lesions caused by systemic diseases; and (3) infectious diseases.

### Ophthalmic artery model reconstruction

**Fig. 1:**
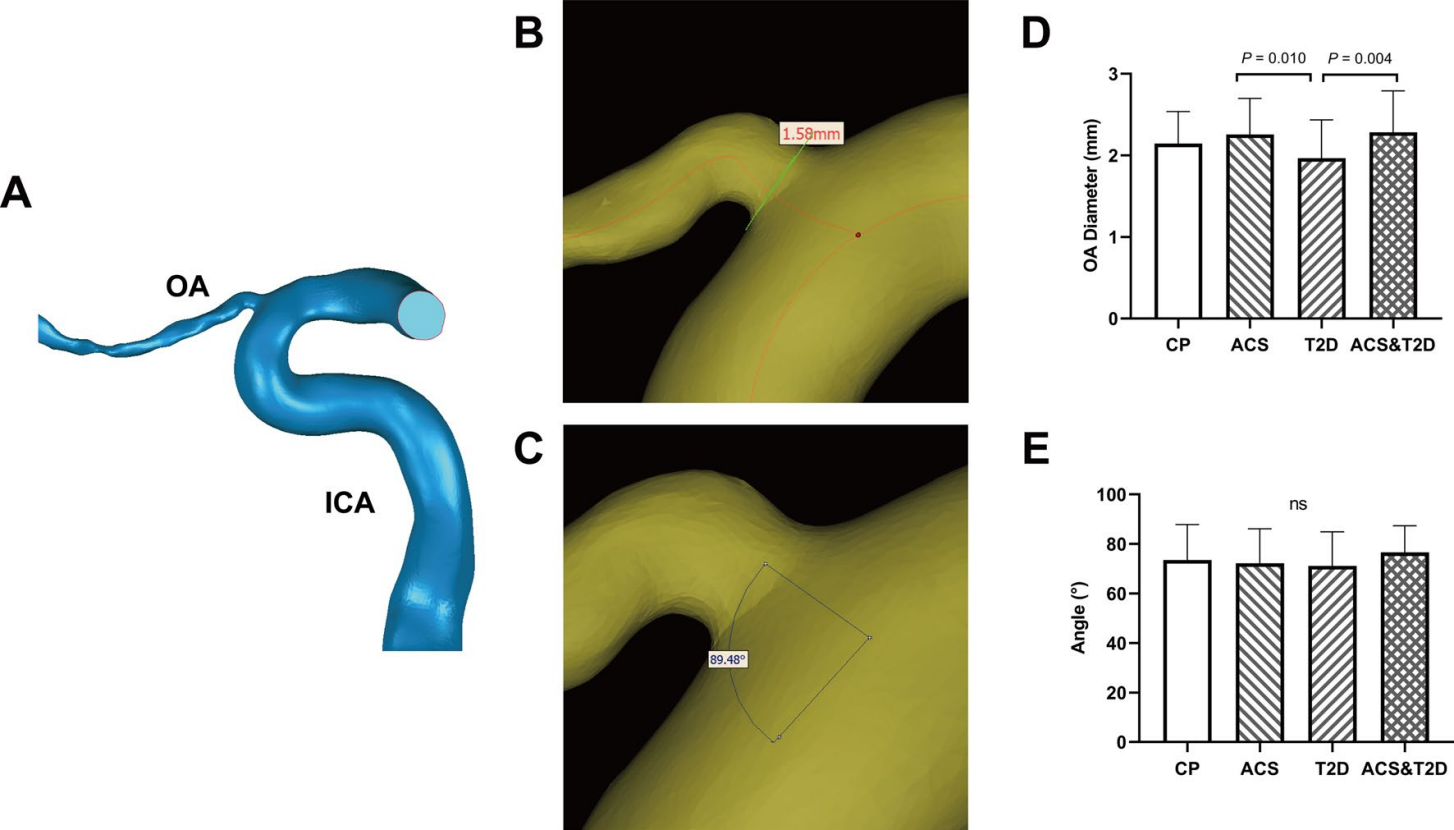
Morphological characteristics of ophthalmic artery (OA). **A** Case example of three-dimensional OA model reconstructed from the computed tomographic angiography images. ICA, internal carotid artery. **B**, **C** Measurement of the diameter and angle between OA and ICA in Mimics (v21.0, Materialise, Ann Arbor, MI, USA). **D**, **E** Comparison of OA diameter and angle. CP, control participants; ACS, acute coronary syndrome; T2D, type 2 diabetes; ns, no significant

The method of OA 3D model reconstruction and blood flow simulation was similar to that used in our previously published study [16]. The Digital Imaging and Communications in Medicine images of the head and neck CTA were imported into Mimics 21.0 (Materialise, Ann Arbor, MI, USA). The OA model of each participant underwent reconstruction. We used image segmentation technology to extract the OA and internal carotid artery (ICA) contours from the CTA images and edit them, manually. Other interfering parts were erased, and we only retained a certain length of the ICA and OA. After the reconstruction, Geomagic Studio 14.0 (3D Systems, Rock Hill, SC, USA) was used for the surface smoothing of OA models and to create solid vessel models (Fig. 1A).

The centerline of each OA model was generated, and the centerline best-fit diameter of the initial OA and the included angle, between the OA and ipsilateral ICA, were measured (Fig. 1B, C). The initial OA was defined as the start of OA branching from the ICA.

### Computational fluid dynamics simulation

ANSYS Fluent 15.0 (ANSYS, Inc., Canonsburg, PA, USA) was used for the blood flow simulation. The finite volume method was adopted, based on a completely unstructured grid. We discretized OA models into tetrahedral and tri-prism mixed elements. We used the SIMPLE algorithm to calculate, and a pressure base was set to correct the pressure and solve the momentum equation, sequentially. The vessels were considered rigid, with no slip, and the simulated blood was assumed to be a steady, laminar, and incompressible Newtonian fluid. The Navier–Stokes and mass conservation equations are the governing equations:

1 $$\rho \left(\overrightarrow{u}\bullet \nabla \right)\overrightarrow{u}+\nabla p-\mu \Delta \overrightarrow{u}=0,$$

2 $$\nabla \bullet \overrightarrow{u}=0.$$

In the formula, $\overrightarrow{u}$ represents the velocity vector, $p$ represents pressure, $\rho$ represents blood density, and μ represents blood viscosity. The blood viscosity and density were set to 3.5 × 10^–3^ kg/ms and 1050 kg/m^3^, respectively. We adopted a systolic and diastolic mean flow velocity of 0.34 m/s as the inlet velocity (velocity of ICA siphon), based on literature[17]. For simplification, we set the outlet as a pressure boundary condition of P = 0 Pa. All models were set the same boundary conditions. In addition, for the numerical simulation and analysis of the fluid part, we set sufficient iterations (0.00001) to obtain a stable solution, after calculation convergence.

After the calculation, the hemodynamic values of the initial OA were recorded from the fluid velocity streamline and pressure contour charts. Following this, we calculated the mass flow of the OA and ICA and the proportion of the ICA flowing into the OA. The results were analyzed by two experienced ophthalmologists.

### Statistical analysis

The analysis was performed using the Statistical Package for the Social Sciences (SPSS) software (version 26.0; SPSS Inc., Chicago, DE, USA). Data was tested for normality using the Shapiro–Wilk test and expressed as mean ± standard deviation. Descriptive data for non-normal distributions are expressed as the median (25–75th percentile). One-way analysis of variance with the Bonferroni correction was used for normally distributed continuous variables, to compare means among groups, and the Kruskal–Wallis *H* test was used for non-normally distributed variables. Comparisons between the two groups were performed using the *t*-test or the Mann–Whitney *U* test. Categorical variables are expressed as numbers and percentages and analyzed using the χ^2^ test or Fisher’s exact test, as appropriate. Pearson’s correlation coefficient and linear regression were used to determine the correlation between continuous variables. Non-normally distributed variables were transformed into natural logarithms.

## Results

### Clinical assessment

Table 1 shows the demographic and comorbidity details of all 134 participants (30 control participants, 34 with ACS only, 34 with T2D only, and 36 with both ACS and T2D). The four groups were matched for age (*P* = 0.099), sex (*P* = 0.089), and history of hypertension (*P* = 0.138). There were no differences in current smoking status (*P* = 0.081) or peripheral arterial disease (*P* = 0.292). A higher proportion of patients with T2D and ACS had a history of dyslipidemia and ischemic stroke (*P* < 0.001 and *P* = 0.007, respectively). The clinical, laboratory, echocardiographic, and medication details are shown in Table 2.

**Table1:** Demographic and comorbidity results in control participants and patients with ACS and T2D

Variables	Control participants (n = 30)	ACS (n = 34)	T2D (n = 34)	ACS and T2D (n = 36)	P value
Age (y), mean ± SD	60.33 ± 5.01	63.12 ± 4.82	62.64 ± 3.81	61.61 ± 5.28	0.099
Female sex, n (%)	16 (53)	8 (24)	14 (41)	12 (33)	0.089
Current smoking, n (%)	8 (27)	14 (41)	11 (32)	20 (56)	0.081
Hypertension, n (%)	16 (53)	24 (71)	20 (59)	28 (78)	0.138
Dyslipidaemia, n (%)	10 (33)	26 (76)	20 (59)	28 (78)	** < 0.001**
PAD, n (%)	4 (13)	8 (24)	8 (24)	12 (33)	0.292
History of ischemic stroke, n (%)	2 (7)	14 (41)	14 (41)	14 (39)	**0.007**

ACS, acute coronary syndrome; T2D, type 2 diabetes; PAD, peripheral arterial disease. P < 0.05 is significant (bold values)

**Table 2:** Clinical, laboratory, echocardiography and medication results in patients with ACS and T2D

Variables	ACS (n = 34)	T2D (n = 34)	ACS and T2D (n = 36)
Clinical characteristics			
BMI (kg/m^2^), mean ± SD	25.46 ± 2.23	25.05 ± 3.00	25.71 ± 2.91
DAC (cm), mean ± SD	90.56 ± 10.11	91.71 ± 6.73	90.94 ± 8.55
Heart rate (bpm), mean ± SD	71.29 ± 12.36	75.00 ± 13.98	74.72 ± 11.81
Systolic BP (mmHg), mean ± SD	138.59 ± 22.40	147.41 ± 19.81	133.39 ± 23.72
Diastolic BP (mmHg), mean ± SD	77.12 ± 14.21	85.35 ± 10.03	76.56 ± 20.00
Laboratory parameters			
TnI (ng/mL), median (IQR 25%–75%)	0.02 (0.00–0.27)	–	0.23 (0.02–1.02)
TnT (ng/mL), median (IQR 25%–75%)	0.01 (0.01–0.39)	–	0.03 (0.01–0.12)
CK (U/L), median (IQR 25%–75%)	141.00 (89.25–249.75)	–	66.50 (56.00–123.00)
CK–MB (ng/mL), median (IQR 25%–75%)	2.30 (1.25–6.78)	–	1.80 (1.10–4.90)
LDH (U/L), median (IQR 25%–75%)	194.00 (168.00–267.50)	–	189.00 (153.00–222.00)
NT–proBNP (pg/mL), median (IQR 25%–75%)	187.00 (83.40–656.25)	–	524.00 (106.00–989.00)
Scr (μmol/L), mean ± SD	64.81 ± 12.92	67.71 ± 16.95	68.93 ± 10.09
HBA1c (%), median (IQR 25%–75%)	6.00 (5.78–6.13)	8.20 (6.55–9.88)	7.60 (6.60–8.50)
FBG (mmol/L), median (IQR 25%–75%)	–	7.20 (6.15–11.43)	6.90 (6.00–11.40)
TC (mmol/L), mean ± SD	4.71 ± 0.85	4.62 ± 1.27	3.38 ± 0.69
TG (mmol/L), mean ± SD	1.43 ± 0.54	1.34 ± 0.50	1.56 ± 0.65
HDL (mmol/L), mean ± SD	1.10 ± 0.24	1.27 ± 0.23	1.07 ± 0.46
LDL (mmol/L), mean ± SD	2.76 ± 0.65	2.57 ± 0.85	1.76 ± 0.52
Sodium (mmol/L), mean ± SD	139.48 ± 1.83	140.53 ± 2.87	140.64 ± 2.59
Potassium (mmol/L), mean ± SD	3.89 ± 0.41	4.16 ± 0.37	4.04 ± 0.37
Echocardiography, mean ± SD			
LVEF (%)	62.91 ± 8.68	–	62.83 ± 8.12
E/A	0.78 ± 0.23	–	0.87 ± 0.22
Cardiac index (L/min/m2)	2.68 ± 0.53	–	2.89 ± 0.49
Concomitant medication, n (%)			
Statin	30 (88)	22 (65)	30 (83)
Aspirin	24 (71)	18 (53)	32 (89)
Clopidogrel/ticagrelor	26 (76)	2 (6)	30 (83)
ACE inhibitor/ARB	8 (24)	10 (29)	18 (50)
Beta blocker	24 (71)	10 (29)	24 (67)
Calcium channel blocker	14 (41)	18 (53)	12 (33)
Insulin	–	16 (47)	10 (28)
ICA stenosis degree, n (%)			
No stenosis	2 (6)	10 (29)	8 (22)
Mild	20 (59)	14 (41)	6 (17)
Moderate	8 (24)	6 (18)	8 (22)
Severe	4 (12)	4 (12)	14 (39)

BMI, body mass index; BP, blood pressure; TnI, troponin I; IQR, interquartile range; TnT, troponin T; CK, creatine kinase; CK-MB, creatine kinase isoenzyme-MB; LDH, lactate dehydrogenase; NT-proBNP, N-terminal pro-B-type natriuretic peptide; Scr, serum creatinine; HBA1c, hemoglobin A1c; FBG, fasting blood glucose; TC, total cholesterol; TG, triacylglycerol; HDL, high-density protein; LDL, low-density protein; LVEF, left-ventricular ejection fraction; E/A, ratio of early to late transmitral flow velocity; ACE, angiotensin-converting enzyme; ARB, angiotensin receptor blocker; ICA, internal carotid artery

Except for four participants in the ACS group, all patients with ACS underwent percutaneous coronary intervention or coronary artery bypass grafting, before the CTA examination. The degree of ICA stenosis in each group are shown in Table 2, and the diagnosis of ICA stenosis was established based on the North American Symptomatic Carotid Endarterectomy Trial [18].

### Morphological characteristics

The OAs from 134 participants were reconstructed. We measured the centerline best-fit diameter of the initial OA and the angle between the OA and ipsilateral ICA. The mean diameters of the initial OA were 2.14 ± 0.39, 2.26 ± 0.44, 1.97 ± 0.47, and 2.28 ± 0.51 mm for control participants, participants with ACS only, participants with T2D only, and participants with both ACS and T2D, respectively. As shown in Fig. 1D, after Bonferroni correction, there was no statistically significant difference among the four groups (*P* = 0.019). Nonetheless, there were statistical differences between the T2D only group and the other two ACS groups in pairwise comparisons (*P* = 0.010 and *P* = 0.004, respectively).

The angles between the OA and ICA were 73.51° ± 14.30°, 72.19° ± 13.94°, 71.17° ± 13.70°, and 76.59° ± 10.76° in each of these groups, respectively. No significant difference was found in the angle among the groups (*P* = 0.341, Fig. 1E).

### Hemodynamic changes

**Fig. 2:**
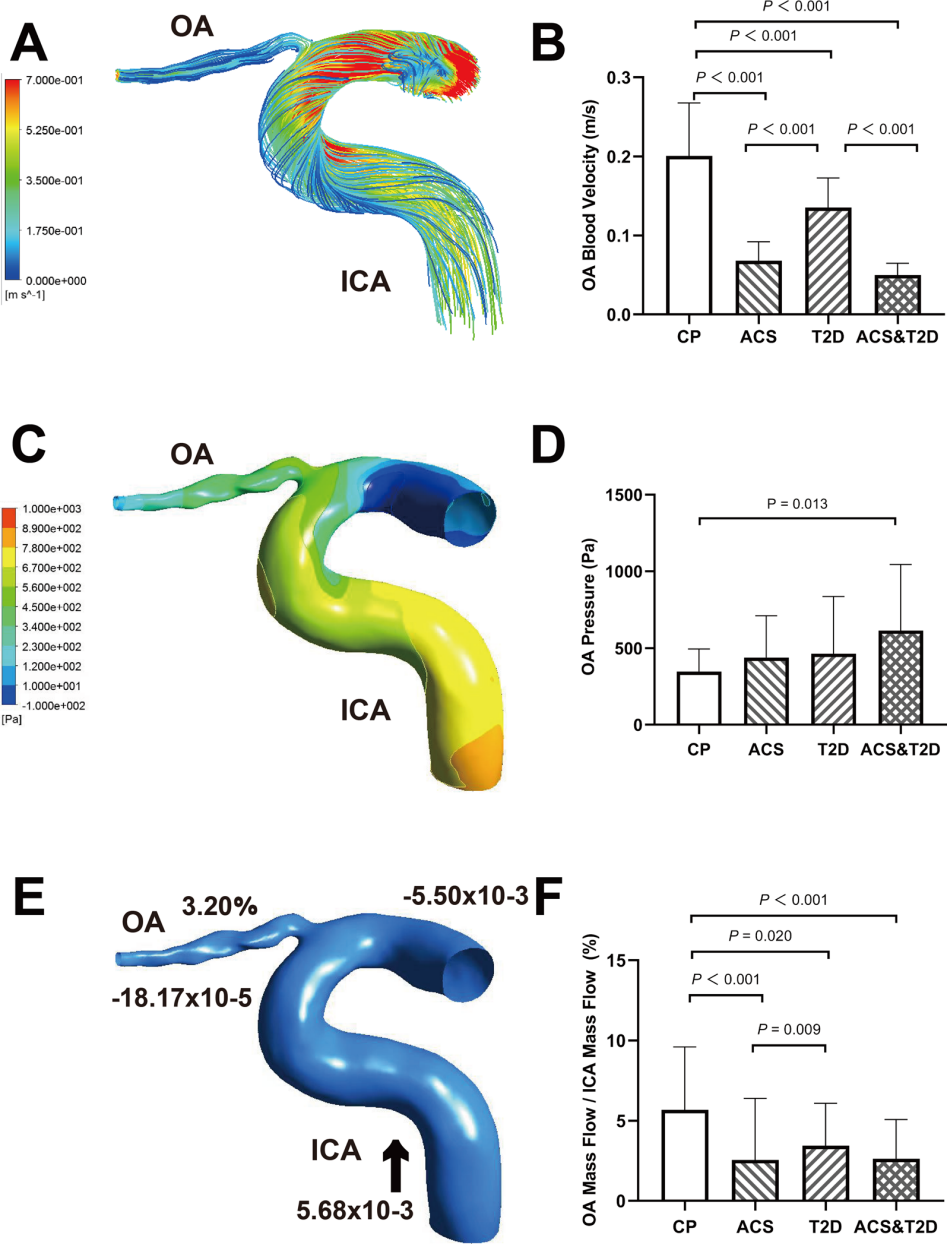
Hemodynamics characteristics of ophthalmic artery (OA). **A** The streamline based on OA blood velocity; **B** Comparison of OA blood velocity. **C** The pressure contour of OA; **D** Comparison of OA pressure. **E** Mass flow (kg/s) and mass flow ratio of OA to ipsilateral internal carotid artery (ICA) (%); **F** Comparison of mass flow ratio of OA to ipsilateral ICA (%). Inlet (+), outlet (−). Blood flow direction (black arrow); CP, control participants; ACS, acute coronary syndrome; T2D, type 2 diabetes

Using CFD numerical simulations, streamline charts were drawn for each OA 3D model (Fig. 2A). Colors in the streamline chart represent different blood flow velocities. Streamlines closer to red indicate higher velocities. Quantitative measurements showed that the blood velocities at origin OA were 0.20 m/s (0.17–0.27 m/s), 0.07 m/s (0.04–0.09 m/s), 0.14 m/s (0.09–0.17 m/s), and 0.05 m/s (0.03–0.06 m/s) in control participants, participants with ACS only, participants with T2D only, and participants with both ACS and T2D, respectively. The statistical differences among the groups are shown in Fig. 2B. The blood flow velocities of the OA in all disease groups were lower than those of the control group (*P* < 0.001). Moreover, patients with T2D only had faster OA blood velocities than the other two ACS groups (*P* < 0.001).

The pressures of the initial OA were 314.63 Pa (246.66–462.00 Pa), 344.63 Pa (209.69–622.79 Pa), 330.57 Pa (198.44–506.10 Pa), and 441.58 Pa (298.78–865.63 Pa) in control participants, participants with ACS only, participants with T2D only, and participants with both ACS and T2D, respectively. Although there was no overall difference among the four groups (*P* = 0.097), we still found that both the ACS and T2D groups had higher OA pressure levels than those of the control group (*P* = 0.013) (Fig. 2C, D).

Similarly, we obtained the mass flow data for each OA model (Fig. 2E). The mass flow ratios of the OA to the ipsilateral ICA were 5.31% (2.49–7.68%), 1.29% (0.84–2.32%), 2.69% (1.38–5.74%), and 1.91% (1.10–3.71%) for control participants, participants with ACS only, participants with T2D only, and participants with both ACS and T2D, respectively. The mass flow ratios in all disease groups were lower than those in the control group (*P* < 0.001, *P* = 0.020, and *P* < 0.001, respectively) (Fig. 2F). In addition, the mass flow ratios of patients with ACS were lower than those of patients with T2D only (*P* = 0.009).

### Association between OA characteristics and clinical parameters

Table 3 displays the correlations between OA characteristics and clinical parameters in patients with ACS and T2D. OA blood flow velocity was positively correlated with diastolic blood pressure and fasting blood glucose, low-density protein, and potassium levels. In contrast, the blood velocity was negatively correlated with left ventricular ejection fraction. In addition, positive correlations were found between the mass flow ratios of the OA to the ipsilateral ICA and troponin I, troponin T, creatine kinase isoenzyme-MB, N-terminal pro-B-type natriuretic peptide (NT-proBNP), hemoglobin A1c, fasting blood glucose, and triacylglycerol levels. There were no correlations between OA morphological indicators (diameter and angle) and clinical parameters.

**Table 3:** Association between OA characteristics and clinical parameters

Variables	ln (Velocity)		ln (Mass flow ratio)	
	Correlation coefficient (r)	P value	Correlation coefficient (r)	P value
Age (y)	− 0.055	0.579	− 0.149	0.131
BMI (kg/m^2^)	− 0.055	0.557	0.056	0.574
DAC (cm)	0.009	0.938	0.133	0.235
Heart rate (bpm)	0.095	0.353	0.112	0.270
Systolic BP (mmHg)	0.152	0.136	0.089	0.381
Diastolic BP (mmHg)	0.209	**0.039**	0.023	0.823
LVEF (%)	− 0.243	**0.042**	− 0.196	0.105
E/A	0.060	0.622	0.108	0.374
Cardiac index (L/min/m^2^)	0.064	0.599	0.078	0.521
ln (TnI) (ng/mL)	− 0.005	0.965	0.311	**0.009**
ln (TnT) (ng/mL)	0.102	0.082	0.497	** < 0.001**
ln (CK) (U/L)	0.082	0.500	0.107	0.379
ln (CK–MB) (ng/mL)	0.037	0.759	0.261	**0.029**
ln (LDH) (U/L)	0.096	0.428	0.121	0.319
ln (NT–proBNP) (pg/mL)	0.069	0.569	0.263	**0.028**
Scr (μmol/L)	0.099	0.320	0.009	0.927
ln (HBA1c) (%)	0.183	0.083	0.333	**0.001**
ln (FBG) (mmol/L)	0.296	**0.004**	0.368	** < 0.001**
TC (mmol/L)	0.161	0.107	0.190	0.055
TG (mmol/L)	− 0.090	0.368	0.233	**0.019**
HDL (mmol/L)	0.015	0.877	0.119	0.232
LDL (mmol/L)	0.199	**0.045**	0.195	0.050
Sodium (mmol/L)	− 0.014	0.886	− 0.166	0.095
Potassium (mmol/L)	0.253	**0.010**	0.142	0.153

BMI, body mass index; BP, blood pressure; TnI, troponin I; IQR, interquartile range; TnT, troponin T; CK, creatine kinase; CK-MB, creatine kinase isoenzyme-MB; LDH, lactate dehydrogenase; NT-proBNP, N-terminal pro-B-type natriuretic peptide; Scr, serum creatinine; HBA1c, hemoglobin A1c; FBG, fasting blood glucose; TC, total cholesterol; TG, triacylglycerol; HDL, high-density protein; LDL, low-density protein; LVEF, left-ventricular ejection fraction; E/A, ratio of early to late transmitral flow velocity; ln, natural log of the variable; P < 0.05 is significant (bold values)

## Discussion

We reported several differences in OA morphology and hemodynamics in patients with ACS only, T2D only, and with both ACS and T2D. It is difficult to obtain hemodynamic data due to the small diameter and complex course of the OA. Most previous studies on OA diameter or blood velocity were based on autopsies [19] or CDI. In comparison, the method in this study could show the full length of the OA and is less affected by human factors.

Nonetheless, in a study that also measured the OA diameter based on CTA [20], the results were different, which may be due to the following reasons. First, the reconstruction methods were different. Second, there was a different measurement method compared to the diameter we measured at the OA’s origin, wherein they measured a diameter of 5 mm from the beginning of the OA. Finally, our control group comprised not completely healthy participants but also matching with the patients in the disease groups in terms of age, sex, smoking history, and hypertension. In our study, the OA diameter of patients with T2D only was smaller than that of patients with ACS, which may be a response of blood vessels to a high glucose status. Consistent with our results, previous studies have reported smaller retinal artery diameters in patients with diabetes. Moreover, arteriole diameter decreased with increasing duration of diabetes, and this difference could not be explained by age, mean arterial blood pressure, or smoking [21, 22]. The main causes of abnormal vascular diameter may be the dysfunction of endothelial cells and abnormal tension of smooth muscle cells and pericytes. Local vasoactive substances play an important role in the regulation of the vascular wall. Additionally, endothelin-1 (ET-1) is a potent endogenous vasoconstrictor. Endogenous expression of ET-1 is increased in diabetes, and ET-1 mRNA levels in patients with diabetes are significantly higher than those in patients without diabetes [23]. ET-1 tends to decrease the retinal artery diameter, but it has no effect on the retinal vein diameter [24]. Notably, ET-1 is a vascular tension regulator of the OA, which can induce strong vasoconstriction and regulate local blood flow [25]. Therefore, it is hypothesized that the increase in ET-1 level is possibly one of the main reasons for the decrease in OA diameter in patients with diabetes. Regarding macrovascularity, studies in recent years have reported its protective effect on aortic aneurysm disease [26, 27], which is associated with the decrease in matrix metalloproteinase activity by high glucose level [28], the non-enzymatic cross-linking of advanced glycation end products between the basement membrane of the extracellular matrix, and the stimulation of the tumor growth factor-β signaling pathway to maintain vascular smooth muscle cell homeostasis [27]. However, studies have found that a smaller retinal artery diameter is associated with the incidence of ACS, especially in women [29], which is inconsistent with the larger OA diameter in our study. This may be affected by the difference in observation time, because, in our study, patients with ACS underwent CTA examination during their hospitalization period. We hypothesize that this is related to the compensatory regulatory mechanism of the ocular blood vessels [25].

In previous studies, CDI has been used to measure retrobulbar blood flow in patients with diabetes. It was found that there was a high resistance index in the OA of patients with diabetes. However, the results of OA blood velocity measurements are controversial. Such inconsistencies may be related to different measurement techniques, locations, and differences in patient population characteristics [9–11]. After blood simulation, we found that the OA blood velocity in patients with ACS was relatively low, which is consistent with the results of our previous study [16]. In this study, the blood velocity in patients with T2D only was lower than that in the control group, but it was higher than that in both ACS groups. This may be due to the increased resistance to outflow from the OA [5]. It is now well established that Cxs plays a vital role in maintaining the activity of microcirculation homeostasis, as well as in pathologies that involve a tight regulation and coordination between cells in the blood vessel wall and circulating blood cells such as atherosclerosis and hypertension [30, 31]. In diabetic retinopathy, breakdown of the blood-retinal barrier is typical and has been associated with pericyte loss and endothelial cellecell junction breakdown, consistent with reports that downregulation of Cx43 may promote vascular loss in the diabetic retina [32]. Müller cell-pericyte communication is critical for maintenance of retinal homeostasis, and that high glucose-induced Cx43 downregulation plays a critical role in the demise of Müller cells and pericytes [33]. Oxidative stress, involved in mitochondrial dysfunction and apoptosis in early damage of diabetic vascular complications [34]. Therefore, maintaining mitochondrial function and attenuating reactive oxygen species production in retinal endothelial cells could be considered a significant approach for treating diabetic retinopathy. Although the potential use of mitochondria-targeted therapies in the regulation of diabetic retinopathy remains unclear, studies have shown that targeting of the mitochondrial superoxide scavenging by overexpression of mitochondrial superoxide dismutase prevents the development of retinopathy in diabetic mice [35, 36]. Intracranial vessels are susceptible to oxidative stress, and destruction of the intracranial carotid blood–brain barrier may precede the formation of atherosclerotic lesions [37]. As a branch of the ICA, OA velocity is easily affected by internal carotid atherosclerotic lesions. In addition, hemodynamic characteristics determine vascular integrity and blood cell transport [38]. Red blood cell deformability was lower in patients with T2D and formed chain-like structures under low shear conditions. Excessive accumulation of red blood cells leads to arteriole blockage, which prevents normal oxygen transport in the surrounding tissues [39]. Notably, the effect of flow perturbation on endothelial cell dysfunction is associated with atherosclerosis [40]. The flow perturbation caused by ACS may also be related to the slowing of the blood flow velocity in the OA. Frank et al. [41]. used CFD to redefine the central role of hemodynamically related microscopic lesions in atherogenesis. They suggested that hemodynamic stress-induced endothelial rupture triggered an inflammatory process that promoted atherosclerosis. This indicates that hemodynamic changes precede pathophysiological changes.

Diabetes causes ischemia in peripheral arterioles, whereas ACS, the acute manifestation of ischemic heart disease, may also affect the blood supply [42]. The incidence of ischemic stroke in patients with diabetes is two to three times higher, due to the combined effect of multiple risk factors for atherosclerosis [37]. Our calculation of the OA mass flow rate showed that there was a decrease in all disease groups, and patients with ACS had a lower flow rate than patients with diabetes. These results further confirm the influence of diabetes and ACS on peripheral blood flow and provide evidence on the pathogenesis of ocular ischemic lesions. Chronic hyperglycemia leading to diabetic retinopathy remains the most common vascular complication in patients with diabetes [43]. Retinal microaneurysm is one of the earliest clinical symptoms of diabetes and one of the key lesions in diabetic retinopathy severity classification. Microaneurysms are local dilations of capillaries. The mechanisms of microaneurysms are unclear, including increased luminal pressure, retinal microenvironment changes, endothelial cell damage due to leukocyte deposition, and pericyte loss [44]. Clinically, patients with ACS are at a higher risk not only for ocular ischemia but also for pressure changes than those with coronary artery disease. Patients with ACS were significantly more likely to develop retinal microaneurysms and dot bleeding than those with stable coronary artery disease [45]. In this study, the OA pressure was higher in patients with both ACS and T2D. We hypothesized that this may increase the risk of dilation, which may further cause lesions, such as microaneurysms and OA aneurysm. It is blood flow that determines aneurysm formation and growth by guiding the collagen remodeling of the aneurysm wall [46]. Pressure-induced fluid changes predate pathophysiological changes [47], and OA pressure changes occur earlier than microaneurysm formation. Therefore, the early observation of hemodynamic changes is important for early clinical detection and treatment.

In the correlation analysis, we found that the OA blood velocity and mass flow ratio were associated with several clinical indicators. Increased fasting plasma glucose and HbA1c levels are important predictors of diabetic complications and are significantly associated with the risk of diabetes, cardiovascular disease, cancer, and all-cause mortality [48]. Although commonly used to assess heart failure, NT-proBNP is independently associated with poor outcomes in patients with myocardial infarction [49]. Meanwhile, the combination of troponin T and NT-proBNP contributes to the identification of patients with diabetes, at an extremely high absolute risk [50]. The hemodynamic parameters of the OA are correlated with important clinical indicators of patient prognosis, which suggests a new study direction for the prediction of disease prognosis.

This study has some limitations. The slice thickness of the CTA scan limits the accuracy of 3D reconstruction. Additionally, the same boundary conditions were applied to all groups because of the lack of relevant study data. Further studies are required to improve the completeness of the results.

## Conclusion

In conclusion, OA 3D models were reconstructed, and blood flow was simulated in patients with T2D, with and without ACS. A finer OA diameter was observed in patients with T2D. In addition, all the disease groups had slow OA blood flow velocity and reduced mass flow, with increased pressure. Moreover, the hemodynamic characteristics of the OA correlated with clinical biomarkers that predict prognosis. Longitudinal studies are required to determine the significance of OA characteristics in the prognosis of ACS and T2D.

## Data Availability

The datasets used and analysed during the current study are available from the corresponding author on reasonable request.

## Abbreviations

3D: Three-dimensional
ACS: Acute coronary syndrome
CDI: Color Doppler imaging
CFD: Computational fluid dynamics
CTA: Computed tomography angiography
Cx: Connexin
ET-1: Endothelin-1
ICA: Internal carotid artery
NT-proBNP: N-terminal pro-B-type natriuretic peptide
OA: Ophthalmic artery
SPSS: Statistical package for the social sciences
T2D: Type 2 diabetes
